# Associations of urological malignancies with renal progression and mortality in advanced chronic kidney disease: a propensity-matched cohort study

**DOI:** 10.1186/s12882-020-01859-w

**Published:** 2020-05-29

**Authors:** Rajkumar Chinnadurai, Noel W. Clarke, Philip A. Kalra

**Affiliations:** 1 Department of Renal Medicine, Salford Royal NHS Foundation Trust, Salford, M6 8HD UK; 2 Faculty of Biology, Medicine and Health, University of Manchester, Manchester, UK; 3 Department of Urology, Salford Royal NHS Foundation Trust, Salford, UK; 4 The Christie NHS Foundation Trust, Manchester, UK

**Keywords:** Urological malignancies, CKD, All-cause mortality, Renal progression

## Abstract

**Background:**

Urological malignancy (UM) in patients with chronic kidney disease (CKD) is an added burden to their overall morbidity and mortality. UM is itself a common cause of CKD. Understanding the associations of UM with outcomes in advanced CKD can help in optimisation of the management of these patients. This study investigates the distribution and association of urological malignancy with outcomes (renal progression and mortality) in patients with advanced non-dialysis dependent CKD.

**Methods:**

The study was conducted in 2637 of 3115 patients recruited in the Salford Kidney Study between the years 2002 and 2016. A comparative analysis was performed between 160 patients with UM (at baseline and incident) and 2477 patients with no malignancy. Cox-regression models and Kaplan-Meir estimates were used to explore the association between the presence of UM with mortality and renal outcome. Linear regression analysis was used to calculate the rate of progression of CKD in the groups. A 1:3 propensity score matched cohort of 640 patients was generated and utilised in the above analyses.

**Results:**

4.4% had a history of UM at baseline with the annual incident rate being 0.37%. The site of malignancy was the kidney in 40% with comparable numbers for prostatic malignancy (39%). 70% (111/160) of UM patients had a medical cause as their primary diagnosis for CKD. Over a median follow up of 4 years, 34% (905) patients died. In the matched sample, the proportion of deaths was similar between the groups (UM 44% versus no malignancy 48%, *p* = 0.36). 30% reached end-stage renal disease (ESRD) with no difference between the groups. In the Cox-regression model, UM did not prove to be a risk factor associated with either all-cause mortality (HR:1.03; CI: 0.79–1.35; *p* = 0.81) or reaching ESRD (HR:1.12; CI: 0.80–1.58; *p* = 0.49). The rate of decline in estimated glomerular filtration rate (eGFR) was similar between the groups (− 1.05 vs − 1.25 mL/min/1.73m^2^/year, *p* = 0.31).

**Conclusions:**

There was no correlation observed between UM and all-cause mortality or ESRD. Medical causes of CKD have a significant influence on the outcomes in patients with UM, whereas the UM did not. Hence, a coordinated approach with early liaison between the urology and nephrology teams is needed in the management of UM patients with CKD.

## Background

Urological malignancies (UM), including malignancies of prostate, kidney, urinary bladder and urinary tract, are highly prevalent in chronic kidney disease (CKD) patients [1]. UM can be the cause or a consequence of CKD. The cause of CKD in patients with UM is often multifactorial, and relevant factors are the site of malignancy (kidneys), urinary tract obstruction and factors related to treatment (chemotherapy, surgery). Several studies have shown an association between CKD and incident UM [1]. Pathogenetic factors have been postulated to include the chronic inflammation, oxidative stress and uremic toxins of CKD as possible triggers [2, 3].

UM in patients with CKD has been shown to be associated with poor prognosis [4, 5]. Further, the post-surgical prognosis of UM is shown to be poor in patients with preoperative CKD due to medical causes [6]. CKD is reported to be a significant risk factor associated with cancer-specific mortality, in particular to cancers of the kidney and urinary tract [7].

Healthcare workers in nephrology and urology work in tandem to prevent the onset and slow the progression of CKD in patients with urological malignancy. Nephrologists are often involved in pre-operative optimisation of UM patients prior to urological intervention, or in follow-up post-intervention. Urologists are in constant pursuit of innovative approaches in the management of these cancers including nephron sparing procedures and robotic surgeries to produce better outcomes. However, the impact of UM malignancies and their management (medical or surgical) on renal outcomes (CKD progression and reaching end-stage renal disease) and consequent overall mortality in advanced CKD (non-dialysis dependent CKD, stage 3–5) patients is still under-explored. Hence, this study aimed to investigate the associations of urological malignancies with mortality and renal outcomes in a large non-dialysis CKD cohort.

## Methods

### Sampling

This study was conducted in Salford Kidney Study (SKS) patients: this is a large prospective CKD cohort recruiting patients since the year 2002. The SKS was previously known as the Chronic Renal Insufficiency Standards Implementation Study (CRISIS). Patient recruitment into SKS has been described in the earlier published literature [8, 9]. Briefly, any adult patient with age > 18 years and eGFR < 60 mL/min/1.73m^2^ referred to the Salford renal service (a tertiary hospital for renal care in the United Kingdom with 1.55 million catchment population) can be approached for consent to the study. Acute kidney injury patients and those who are in immediate need for commencing renal replacement therapy (RRT) are excluded from enrolment in this study as the principal objective of the SKS was to study the cardiovascular disease outcomes during longitudinal follow-up of CKD patients. At study baseline, demographics, comorbidities including the history of cardiovascular events and malignancy, clinical variables and concurrent medications are collected. Baseline blood results including full renal profile, full blood count and bone profile are also recorded. The patients are then followed up on an annual basis to update their comorbidities, hospital admissions, medications and blood results. Data is collected and updated by a dedicated team of research nurses.

**Fig. 1:**
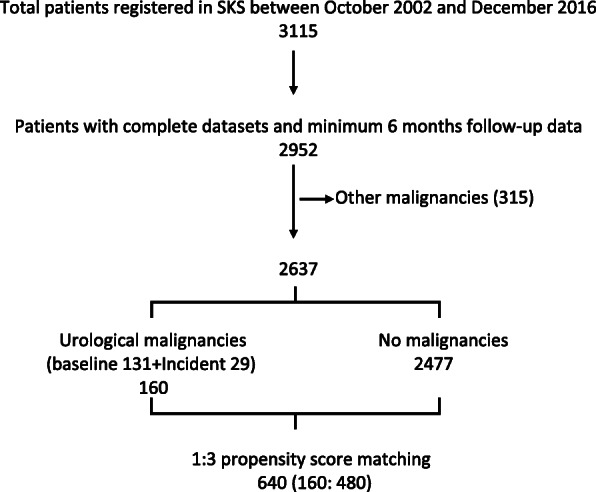
Flowchart of patient recruitment to the study

Of the 3115 patients enrolled in SKS between October 2002 and December 2016, 2952 patients had a complete dataset and minimum 6 months follow-up data. After excluding patients with all other malignancies (both prevalent and incident), 2637 patients were included in this study. From this sample, 160 patients with a history of urological (kidney, prostate, bladder and urinary tract) malignancies were identified (131 at baseline and 29 incident cases during follow-up). A comparative analysis was performed between patients with urological malignancy (160) and those without any malignancy (2477). A 1:3 propensity score matched sample of 640 patients (urological malignancy 160 patients: no malignancy 480 patients) was produced and used for similar analysis. A flowchart illustrating patient recruitment to the study is shown in Fig. 1.

### Data collection

Patients were followed up from study entry (baseline) to endpoints which included commencing RRT, death, lost to follow up or study end date of 31st December 2017. For patients with incident urological malignancy, the study visit before their cancer diagnosis was used as the study baseline and patients were followed-up until the above endpoints had been reached. Baseline blood results included tests undertaken at the baseline date or within 3 months of study entry date. For calculating the rate of decline in CKD, eGFR results only from outpatient clinic visits were used, and only patients with a minimum of three eGFR measurements were included in this analysis.

### Study definitions

A smoking history was defined as a history of smoking irrespective of the number of cigarettes smoked, similarly an alcohol history was defined as alcohol intake irrespective of the number of units of alcohol. A comorbidity of hypertension was defined as a history of hypertension recorded in the hospital or general practitioner’s records, and/or receiving antihypertensive therapy. End-stage renal disease (ESRD) was defined as reaching renal replacement therapy (RRT) or eGFR of < 10 mL/min/1.73m^2^ (to capture patients opting for conservative care). RRT included haemodialysis, peritoneal dialysis and transplantation. Non-fatal cardiovascular events (NFCVE) included a composite of non-fatal cardiac arrest, acute coronary syndrome, myocardial infarction, peripheral vascular disease, cerebrovascular accident and congestive cardiac failure (new diagnosis or hospital admission with exacerbations). Coding for primary renal diagnosis for CKD was based on the European Renal Association and European Dialysis and Transplant Association (ERA-EDTA) coding system. The cause of death data was obtained from the death certificate obtained from the Office of National Statistics and from electronic patient records (EPR).

### Statistics

Statistical analysis was performed using SPSS version-23, licenced to the University of Manchester. Throughout the analysis, categorical values were expressed as number (%), and the *p*-value was derived using the Chi-square test. Most of the data in SKS is non-normally distributed hence for uniformity, continuous values were expressed as median (interquartile range) and the Mann-Whitney U test was used to calculate the *p*-value. A *p*-value < 0.05 was considered statistically significant in this study. Univariate and multivariate Cox-regression models were used to study the association between the presence of urological malignancy, all-cause mortality and reaching end-stage renal disease. To overcome competing risk the follow-up time was censored at the first occurring event in these models [10]. Kaplan-Meier curves were also used for a visual demonstration of these associations, with the log-rank test used for statistical significance in this estimate. Linear regression analysis was used to generate the annual rate of change in eGFR (delta GFR). A competing risk analysis (CRA) for RRT and death between the groups was also performed using the ‘cmprsk’ and ‘survival’ packages in R software, version 3.5.1 [11, 12]. A *p*-value for the CRA was calculated by the modified X^2^ statistic outlined in Gray, 1988 [13]. Patients with urological malignancies were matched with those without any malignancy using propensity score matching. Matching was undertaken for the four major variables that were significantly different between the groups: age, gender, ethnicity and smoking status. The groups were matched 1:3 using the neighbour match of patients with the same propensity score, generated by `MatchIt’ package of the R software version 3.5.1 [14].

## Results

**Fig. 2:**
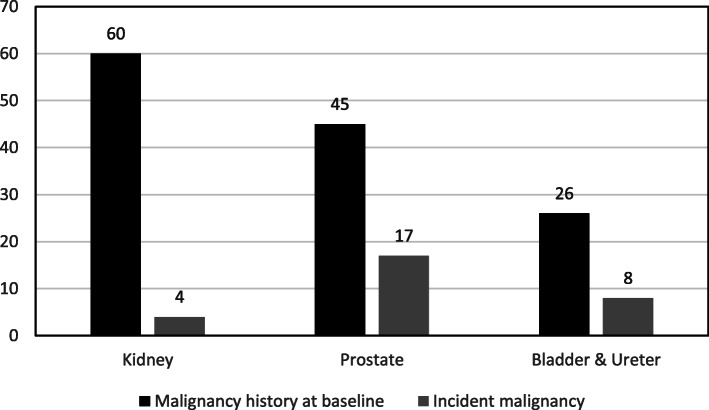
Urological malignancy site distribution

**Fig. 3:**
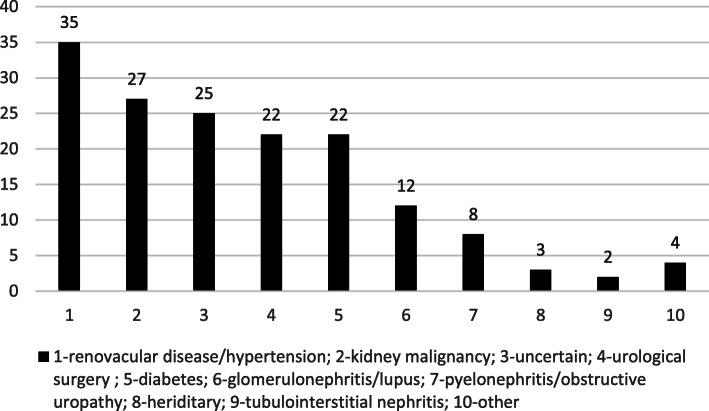
Coded primary renal diagnosis in patients with urological malignancy

At baseline, 4.4% (131/2952) of our cohort had a history of UM (prior UM or current UM at study recruitment into the cohort) and the annual incident rate was 0.37%. Renal malignancy was the most common prevalent site and prostate the commonest incident site (Fig. 2). Management options for UM were found to be variable ranging from radical resection to routine surveillance depending on the site. 61/160 patients had unilateral nephrectomy (partial or total), and 10/160 had radical cystoprostatectomy (Additional file 1: Table S1). The coded primary renal diagnosis for CKD in UM patients is illustrated in Fig. 3. 30% of UM patients had a primary diagnosis of kidney tumour and/or urological surgery while the remaining 70% (111/160) of UM patients had a medical cause as their primary diagnosis for CKD.

The median age of our sample was 67 (interquartile range 55 to 75) years. Our cohort had a predominance of males (63%) and Caucasians (96%). A higher percentage of patients with UM smoked compared to those without any malignancy (72.5% versus 64.7%, *p* < 0.05). 33% of the patients in our cohort had diabetes, with 90% having a history of hypertension. The baseline cardiovascular risk factor profile of the groups was similar. The groups were matched in respect to other baseline characteristics apart from more patients in the no malignancy group having chronic obstructive pulmonary disease (18.5% versus 11.8%, *P* = 0.03) and more receiving renin-angiotensin system (RAS) blockers (62.5% versus 43.8%, *p* < 0.001). Patients with UM had a higher creatinine leading to a lower eGFR (27.7 vs 30.3, *p* < 0.05). Once matched by propensity scores, the groups were similar in most of the baseline characteristics and blood results (Table 1).

**Table 1:** Comparison of baseline characteristics between patients with urological malignancy and no malignancy (total and matched sample)

		Total sample				Matched sample		
Variable	Total (2637)	Urological malignancy (UM) (160)	No malignancy (NM) (2477)	*p*-Value UM vs NM	Total (640)	Urological malignancy (UM) (160)	No malignancy (NM) (480)	*p*-Value UM vs NM
Age, years	67 (55–75.5)	72.5 (66.6–77.4)	66.5 (54.3–75.3)	**< 0.001**	72.5 (66.6–77.5)	72.5 (66.6–77.4)	72.4 (66.6–77.5)	0.97
Gender, male	1652 (62.6)	138 (86.3)	1514 (61.1)	**< 0.001**	550 (85.9)	138 (86.3)	412 (85.8)	0.90
Caucasian	2524 (95.7)	159 (99.4)	2365 (95.5)	**0.02**	638 (99.7)	159 (99.4)	479 (99.8)	0.41
Smoking	1719 (67.2)	116 (72.5)	1603 (64.7)	**0.04**	478 (74.7)	116 (72.5)	362 (75.4)	0.46
Alcohol	1236 (46.87)	77 (48.1)	1159 (46.79)	0.74	315 (49.2)	77 (48.1)	238 (49.6)	0.75
BMI^a^, kg/m^2^	28.1 (24.7–32.6)	28.1 (25.7–32.3)	28.1 (24.6–32.6)	0.55	28.1 (25–32)	28 (25.7–32.3)	28.2 (24.9–32)	0.48
Systolic BP, mmHg	138 (124–152)	140 (130–154)	138 (123–152)	0.08	140 (128–155)	140 (130–154)	140 (127–155)	0.92
Diastolic BP, mmHg	75 (66–80)	75.5 (66–82)	75 (66–80)	0.26	72 (65–80)	75 (66–82)	72 (64–80)	**0.02**
Hypertension	2397 (90.9)	140 (87.5)	2257 (91.1)	0.12	579 (90.5)	140 (87.5)	439 (91.5)	0.14
DM	866 (32.8)	53 (33.1)	813 (32.8)	0.94	244 (38.1)	53 (33.1)	191 (39.8)	0.13
IHD	492 (18.65)	34 (21.3)	458 (18.5)	0.39	46 (7.2)	34 (21.3)	112 (23.3)	0.59
MI	414 (15.7)	33 (20.6)	381 (15.4)	0.08	150 (23.4)	33 (20.9)	117 (24.4)	0.33
CCF	454 (17.2)	25 (15.6)	429 (17.3)	0.58	128 (20)	25 (15.8)	103 (21.5)	0.11
CVA	214 (8.1)	16 (10)	198 (7.99)	0.37	66 (10.3)	16 (10)	50 (10.4)	0.88
PVD	347 (13.2)	18 (11.3)	329 (13.3)	0.46	106 (16.6)	18 (11.4)	68 (14.2)	0.35
COPD	478 (18.1)	19 (11.8)	459 (18.5)	**0.03**	113 (17.6)	19 (12.1)	94 (19.6)	**0.03**
CLD	79 (2.9)	1 (0.63)	78 (3.1)	0.07	12 (1.87)	1 (0.63)	11 (2.29)	0.18
RAS blocker	1618 (61.4)	70 (43.8)	1548 (62.5)	**< 0.001**	355 (55.5)	70 (44.3)	285 (59.3)	**0.001**
Statin	1545 (58.6)	95 (59.4)	1450 (58.5)	0.84	415 (64.8)	95 (59.4)	320 (66.6)	0.09
ESA	340 (12.9)	14 (8.8)	326 (13.2)	0.11	84 (13.1)	14 (8.9)	70 (14.6)	0.06
Hb, g/L	123 (112–136)	124 (113–137)	123 (112–135)	0.67	123 (112–135)	124 (113–137)	123 (112–135)	0.57
ALP, Units/L	82 (65–103)	83 (66–100)	81 (65–103)	0.67	82 (66–102)	83 (66–101)	81 (66–102)	0.64
Calcium, mmol/L	2.30 (2.21–2.39)	2.31 (2.22–2.37)	2.30 (2.21–2.39)	0.91	2.30 (2.20–2.37)	2.31 (2.22–2.37)	2.30 (2.20–2.37)	0.28
Phosphate, mmol/L	1.12 (0.97–1.28)	1.09 (0.93–1.26)	1.12 (0.97–1.29)	**0.04**	1.07 (0.94–1.25)	1.09 (0.93–1.26)	1.07 (0.94–1.25)	0.96
uPCR^b^, g/mol	31 (13–107)	32.6 (12.9–93.4)	31.33 (13.04–107.7)	0.63	25.3 (12.5–85.3)	32.6 (12.9–88.2)	24.4 (12.4–82.8)	0.37
Creatinine, micromol/L	181 (135–256)	197 (151–286)	179 (135–254)	**0.004**	190 (145–274.7)	197 (151–286)	189 (144–272)	0.25
eGFR, mL/min/1.73m^2^	30 (20–42.9)	27.7 (17.5–39.1)	30.3 (19.8–43.2)	**0.03**	28.6 (18.1–39.8)	27.75 (17.5–39.1)	28.8 (18.5–40.1)	0.41

*BMI* body mass index, *BP* blood pressure (mm of Hg), *DM* diabetes mellitus, *IHD* ischemic heart disease, *MI* myocardial infarction, *CCF* congestive cardiac failure, *CVA* cerebrovascular accident, *PVD* peripheral vascular disease, *COPD* chronic obstructive pulmonary disease, *CLD* chronic liver disease, *RAS* renin-angiotensin system, *ESA* erythropoietin stimulating agents, *Hb* haemoglobin, *ALP* alkaline phosphatase, *uPCR* urine protein creatinine ratio, *eGFR* estimated glomerular filtration rate calculated by CKD-EPI equation. Continuous variables are expressed as median (interquartile range) and *p*-Value by Mann-Whitney U test

Categorical variables are expressed as number (%) and *p*-Value by Chi-Square test

^a^BMI missing in 469 of 2477 of total sample and in 112 of 640 of matched sample

^b^Missing uPCR values in 265 patients of total sample and 66 patients in matched sample

A total of 905 (34%) patients died over a median follow-up of 48 months. The all-cause mortality was noted to be higher in the UM group (43.8% versus 33.7%, *p* = 0.01), but this difference disappeared once the groups were matched. The cause of death data was available only in 52.6% (476/905) of the total number of deaths. Cancer-specific mortality was the leading cause of death in the UM group, while cardiovascular death was the leading cause in the no malignancy CKD group. However, the age at death was not significantly different between the groups (matched sample *p* = 0.56). There was no difference observed between the groups with regard to reaching ESRD (28.1% versus 29.5%, *p* = 0.70) and the uptake of RRT showed no difference (17.5% versus 21.8%, *p* = 0.20). Further, the CKD progression as determined by the linear regression analysis showed that the annual rate of decline in eGFR was similar between the groups (− 1.05 versus − 1.25 mL/min/1.73m^2^, *p* = 0.31). Also, the number of NFCVE were similar between the groups in both the total and matched sample (Table 2).

**Table 2:** Comparison of mortality and renal outcomes between patients with urological malignancy and no malignancy in both total and matched sample

	Total sample				Matched sample		
Variable	Total (2637)	Urological malignancy (160)	No malignancy (2477)	*p*-Value	Urological malignancy (160)	No malignancy (480)	*p*-Value
Follow up, months	48 (25.5–79)	40.6 (23.4–69.6)	48.6 (25.6–79.5)	**0.02**	41.3 (23.7–72.7)	47.4 (25.8–77.8)	0.22
All-cause mortality	905 (34.3)	70 (43.75)	835 (33.7)	**0.01**	70 (43.8)	230 (47.9)	0.36
1st Cause death (Malignancy)	11/476 (2.3)	11/40 (27.5)	0/436 (0)	**< 0.001**^**a**^	11/40 (27.5)	0/111	**< 0.001**^**a**^
1st Cause death (CVD)	179/476 (37.6)	7/40 (17.5)	172/436 (39.4)	**0.006**	7/40 (17.5)	53/111 (47.7)	**< 0.001**
1St Cause death (Infection)	142/476 (29.8)	10/40 (25)	132/436 (30.3)	0.49	10/40 (25)	29/111 (26.1)	0.89
1st Cause death (ESRD)	55/476 (11.6)	4/40 (10)	51/436 (11.7)	1.00^a^	4/40 (10)	9/111 (8.1)	0.75^a^
Age at death, years	78.7 (72–84.4)	79.4 (73–83.8)	78.6 (72–84.4)	0.50	78.8 (72.9–83.7)	79.4 (74.5–83.6)	0.56
ESRD	777 (29.5)	45 (28.1)	732 (29.5)	0.70	45 (28.1)	131 (27.3)	0.84
RRT	568 (21.5)	28 (17.5)	540 (21.8)	0.20	28 (17.7)	72 (15)	0.45
NFCVE	251 (9.5)	17 (10.6)	234 (9.4)	0.24	17 (10.6)	59 (12.29)	0.57
Rate of decline of eGFR (eGFR slope) mL/min/1.73m^2^/year		−1.05 (−2.5 to 0.52)	−1.25 (−3.27 to 0.51)	0.31	−1.05 (−2.5 to 0.52)	−0.88 (− 2.3 to 0.61)	0.58

Cause of death represents 1a cause of death in death certificate. Cause of death available only in 476/905 patients of the total sample and 151/300 patients of the matched sample. CVD- cardiovascular disease, RRT- renal replacement therapy, ESRD- end-stage renal disease, NFCVE- non-fatal cardiovascular events

Continuous variables are expressed as median (interquartile range) and *p*-Value by Mann-Whitney U test

Categorical variables are expressed as number (%) and *p*-Value by Chi-square test

eGFR -estimated glomerular filtration rate calculated by CKD-EPI equation

eGFR slope was calculated on 2459/2637 patients in total sample and 593/640 patients in matched sample with three or more eGFRs

^a^*p*-Value by Fisher exact test

**Fig. 4:**
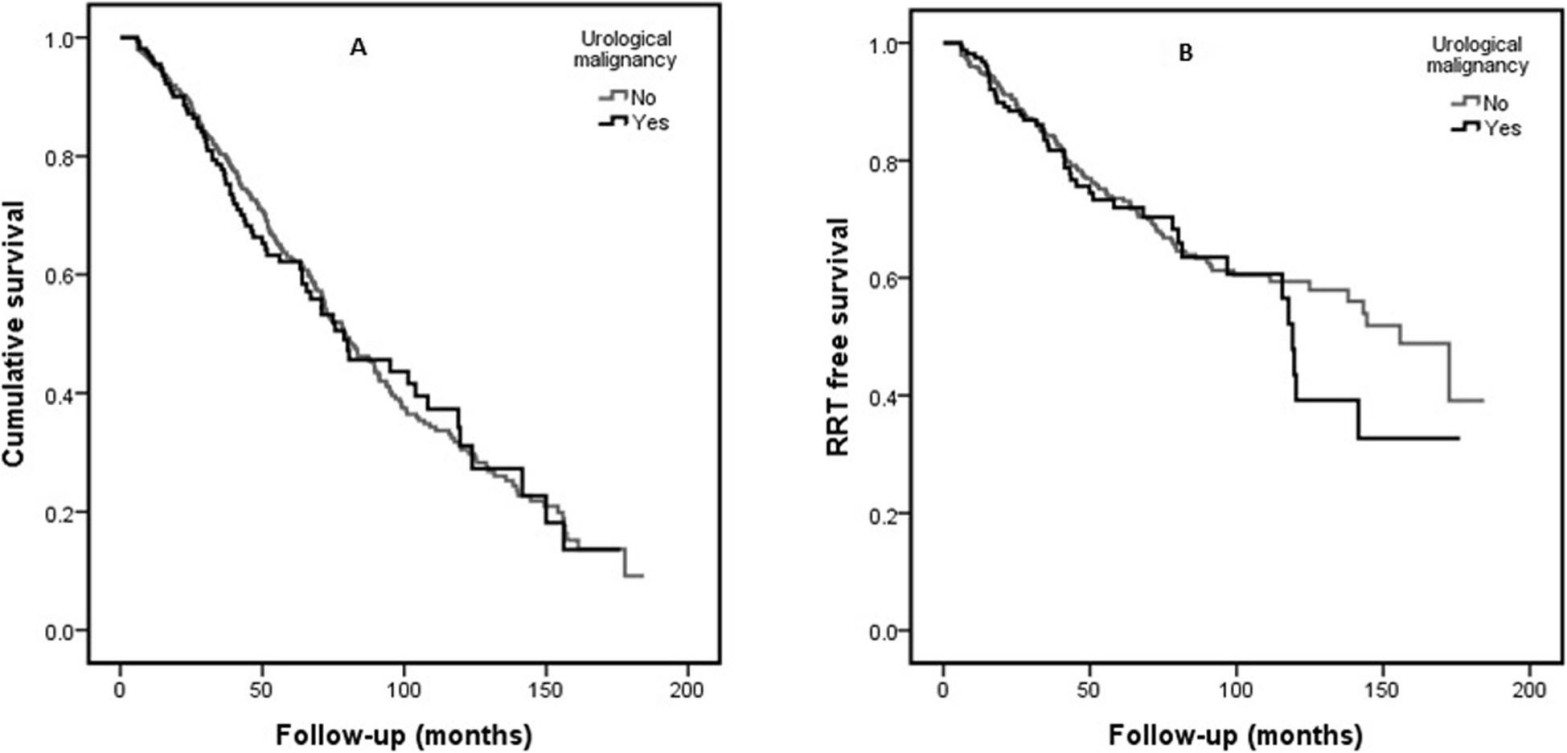
Kaplan-Meier survival curve in the matched sample (**a**: all-cause mortality and panel **b**: RRT free survival)

The univariate Cox-regression model showed a strong association between the presence of urological malignancy and all-cause mortality (HR:1.62, *p* < 0.001). However, the significance was lost in the multivariate model that was adjusted for age, gender and ethnicity (HR; 1.26, *P* = 0.06). Similarly, there was no association noted in the matched sample even in the univariate model (HR: 1.03, *p* = 0.81) (Table 3 and Additional file 1: Table S2). The Kaplan-Meier curve for all-cause mortality showed no survival difference between the groups in the matched sample (Log-Rank test: *p* = 0.81) (Fig. 4a).

**Table 3:** Association of urological malignancy with all-cause mortality and reaching end-stage renal disease (Cox regression models)

	Total sample	*p*-Value	Matched sample	*p*-Value
	HR (95% CI)		HR (95% CI)	
**Urological malignancy and all-cause mortality**				
Univariate model	1.62 (1.27–2.07)	**< 0.001**	1.03 (0.79–1.35)	0.81
Multivariate model-1	1.26 (0.98–1.62)	0.06	1.05 (0.81–1.38)	0.70
**Urological malignancy and end-stage renal disease**				
Univariate model	1.08 (0.80–1.47)	0.59	1.12 (0.80–1.58)	0.49

Multivariate Model-1: Adjusted for age, gender and ethnicity

eGFR - estimated glomerular filtration rate calculated by CKD-EPI equation

*HR* Hazard ratio, *CI* Confidence interval

There was no correlation between the presence of urological malignancy and reaching ESRD. (Table 3 and Additional file 1: Table S3). Lack of correlation in reaching ESRD is also shown in the Kaplan-Meier curve by RRT free survival in patients with and without urological malignancy (Log-rank; *p* = 0.49) (Fig. 4b).

In the competing risk analysis, the cumulative incidence of RRT was similar between the groups both at 5 years (0.16 versus 0.19) and at 10 years (0.22 versus 0.26), *p* = 0.279, and the probability of reaching RRT also reflected this in the matched sample. In contrast, the cumulative incidence for death was noted to be greater in the urological malignancy group both at five (0.36 vs 0.24) and 10 years (0.57 versus 0.44), *p* = < 0.001. The difference between the groups in the cumulative incidence of death was not observed in the matched sample (Table 4).

**Table 4:** Comparison of cumulative incidence of renal replacement therapy (RRT) and death at 5 and 10 years follow up between patients with urological malignancy and no malignancy in a competing risk model

		Total sample			Matched sample		
Event	Time	Urological malignancy (160)	No malignancy (2477)	*p*-Value	Urological malignancy (160)	No malignancy (480)	*p*-Value
RRT	5 years	0.16	0.19		0.16	0.13	
	10 years	0.22	0.26	0.28	0.22	0.18	0.30
Death	5 years	0.36	0.24		0.36	0.33	
	10 years	0.57	0.44	**< 0.001**	0.57	0.59	0.81

RRT-renal replacement therapy (haemodialysis, peritoneal dialysis and transplant)

Values represent cumulative incidence probability for events. *p*-Value by X^2^ statistics (Gray,1988)

The sub-analysis of 160 patients with UM showed that, 47 patients had a history of malignancy more than 5 years from recruitment into the cohort (group A), 61 had a history of malignancy with diagnosis within 5 years of recruitment (group B), and 52 had concurrent and incident malignancy (group C). The CKD status before the onset of malignancy was not available for the 68% of patients with prior history of UM. There was no significant difference in the proportion of deaths between these 3 groups (*p* = 0.44). Similarly, the proportion of patients reaching ESRD and RRT uptake was similar between the groups (Table 5).

**Table 5:** Comparison of outcomes between the groups split based on date of cancer occurrence prior to recruitment and incident cancer

Outcome	No malignancy (480)	Malignancy history > 5 years Group A (47)	Malignancy history < 5 years Group B (61)	Concurrent/incident malignancy Group C (52)	*p*-Value A vs C
Death	230 (48%)	19 (40.4%)	26 (42.6%)	25 (48.1%)	0.44
Malignancy death	0/111	1/12 (8.3%)	5/14 (35.7%)	5/14 (35.7%)	0.17^a^
CVD death	53/111 (47.7%)	1/12 (8.3%)	3/14 (21.4%)	3/14 (21.4%)	0.59^a^
ESRD	131 (27.3%)	13 (27.7%)	17 (27.9%)	15 (28.8%)	0.89
RRT	72 (15%)	11 (23.4%)	9 (14.8%)	8 (15.4%)	0.31
Renal transplants	18 (13.7%)	1/13 (7.7%)	1/17 (5.9%)	2/15 (13.3%)	1.00^a^

Cause of death available only in 151/300 of matched sample

*CVD* cardiovascular disease, *ESRD* end-stage renal disease, *RRT* renal replacement therapy

*p*-Value by Chi-square test. ^a^*p*-Value by Fisher exact test

Kaplan Meier (KM) analysis of the subgroups (no malignancy, > 5 years, < 5 years, concurrent and incident) showed no distinction between the groups in survival outcomes (log-rank, *p*-Value = 0.324) (Additional file 2: Figure S1A). In a KM chart of outcomes of patients with different urological cancer sites in the matched sample, there was an overlap of the survival pattern. (log-rank, *p* = 0.278) (Additional file 2: Figure S1B).

## Discussion

Several studies have cited the presence of CKD as a poor prognostic marker for patients with urological malignancy [15, 16]. The prevalence (4.4%) and annual incident rates (0.57%) of urological malignancies in our cohort was comparable to other observational studies in CKD patients [2, 17]. The higher prevalence and low incidence of kidney cancers in our cohort probably reflects patients being referred to the nephrology service post-operatively for ongoing CKD monitoring. The coded primary diagnosis for the majority (70%) of patients with UM was a medical cause, suggesting a multifactorial aetiology for their CKD progression. Patients with UM were older compared to those without, and age-related association with cancer is well documented [18]. There were more males in the UM group (86.3% versus 61.1%, *p* < 0.001), likely due to the inclusion of prostate cancer. More patients in the UM group had a history of smoking and cigarette smoking is well known to be associated with genitourinary malignancy [19]. The propensity score matching produced a well-matched sample with minimal difference in these baseline or biochemical characteristics.

The median follow-up duration was significantly lower in the group with UM (40.6 versus 48.6 months, *p* = 0.02), probably due to their older age at recruitment. Loss to follow-up is one of the endpoints of the Salford Kidney Study, but this did not affect the results of this study as only small numbers were lost to follow up (*n* = 87). All-cause mortality rate was higher in the UM group mainly attributed to malignancy specific death; however cardiovascular disease-related mortality was low compared to the no malignancy CKD group (17.5% versus 39.4%, *p* = 0.006). The United States Renal Data System (USRDS) showed similar data in ESRD patients with renal malignancy, i.e. a higher cancer-specific mortality and a lower non-cancer specific mortality [20]. Renal outcomes including reaching ESRD and the RRT uptake were not different between the groups. In a study by Lee et al. in patients who had radical nephroureterectomy for upper tract urothelial carcinoma, those with low eGFR (< 60 mL/min/1.73m^2^) pre-operatively were more likely to experience complete recovery of renal function compared to patients with higher eGFR, and although counter-intuitive this was proposed to be due to compensatory hypertrophy of the surviving kidney [21].

In our cohort, urological malignancy did not show an independent association with all-cause mortality. A Japanese CKD cohort with a predominance of urological malignancies had similar findings, with the presence of malignancy associated with malignancy-associated mortality (HR: 3.94; 95% CI: 1.63–9.53; *P* = 0.002) but not all-cause mortality (HR: 1.34; 95% CI: 0.72–2.52; *P* = 0.35) [17]. However, our observational study included a much larger sample size compared to the Japanese cohort (2637 versus 515 patients). Kim et al. also developed a propensity score matched cohort study of Korean patients with renal cell carcinoma and showed that the presence of pre-operative CKD had a strong association with cancer-specific mortality (HR:1.62; *p* = 0.02), but the association to all-cause mortality was close to losing significance (HR:1.45; *p* = 0.049) [22].

The presence of urological malignancy was not associated with an increased risk of reaching ESRD and it did not accelerate the rate of progression of CKD (eGFR slope similar in both groups in the matched sample). In a meta-analysis of 58 studies, Patel et al. showed the overall rate of ESRD to be low (0.4–2.8%) irrespective of the management strategy for renal cell carcinoma [23]. Moreover, a lower rate of renal functional decline was noted in patients with surgically induced CKD than in those patients with CKD from a medical cause who then underwent surgery [24].

In competing risk models, the cumulative probability of death was higher for patients in the UM group both at 5 years and 10 years. However, there was no difference in the cumulative incidence of progression to RRT. Demirjian et al. undertook a competing risk model in renal cancer surgery patients and showed that surgical CKD had a better prognosis than a combination of medical and surgical CKD in terms of renal function decline and mortality [25].

Our study is a single centre analysis of predominantly Caucasian patients who volunteered for participation in the cohort, and this warrants caution with respect to generalising the results to the entire CKD population. Non-availability of the CKD status of all the UM patients before the onset of malignancy restricted our ability to delineate the exact influence of CKD on the outcomes in patients with malignancy. Also, a large proportion of missing cause of death data limited the strength of investigating the association of UM with cancer-specific mortality. Despite these limitations, our study strengths included a robust database with large sample size and adequate follow-up data.

## Conclusion

In conclusion, in our cohort of patients with advanced CKD, the presence of UM was not found to be associated with all-cause mortality. In addition, and independent of the management approach, the presence of UM did not prove to be a risk factor associated with worse renal outcomes (acceleration of the rate of CKD progression or reaching ESRD). Our study shows that the underlying medical causes of CKD, including diabetes and hypertension, may be the predominant influencers of the outcomes (renal and mortality) in patients with urological malignancies. A coordinated approach with early liaison between the urology and nephrology teams is needed in the management of UM patients with CKD in view of this, as the presence of UM did not appear to influence outcome. Future research to compare the outcomes in UM patients with and without the traditional cardiovascular risk factors including CKD can shed light on the risk stratification of these patients.

## Supplementary information

**Additional file 1: Table S1.** Management strategies according to site of malignancy. **Table S2**. Association of urological malignancy with all-cause mortality (Cox regression analysis- univariate model). **Table S3**. Association of urological malignancy with end-stage renal disease (Cox regression analysis- univariate model).

**Additional file 2: Figure S1**. Kaplan-Meier curve for all-cause mortality in the matched sample (A: comparison between groups split based on date of cancer occurrence prior to recruitment; B: comparison between groups split based on site of cancer).

## Data Availability

The authors would not like to share the data as this study derives from a precious long-standing database in which data has been meticulously collected over 20 years. The authors are shortly planning to perform further analyses from the data, and these would be compromised if the database were made publically available but are available from the corresponding author on reasonable request.

## Abbreviations

CKD: Chronic kidney disease
CRA: Competing risk analysis
EPR: Electronic patient records
ESRD: End-stage renal disease
eGFR: Estimated glomerular filtration rate
KM: Kaplan Meier
NFCVE: Non-fatal cardiovascular events
RAS: Renin-angiotensin system
RRT: Renal replacement therapy
SKS: Salford Kidney Study
UM: Urological malignancy
