# Supplementary material for: Associations of urological malignancies with renal progression and mortality in advanced chronic kidney disease: a propensity-matched cohort study
Source: BMC Nephrol. 2020 May 29;21:202. doi: 10.1186/s12882-020-01859-w (PMC7257121; doi:10.1186/s12882-020-01859-w)
Supplement: Supplementary file 1 — Additional file 1: Table S1. Management strategies according to site of malignancy. Table S2. Association of urological malignancy with all-cause mortality (Cox regression analysis- univariate model). Table S3. Association of urological malignancy with end-stage renal disease (Cox regression analysis- univariate model). [file 12882_2020_1859_MOESM1_ESM.docx]

**Associations of urological malignancies with renal progression and mortality in advanced chronic kidney disease: A propensity-matched cohort study**

**Table S1 Management strategies according to site of malignancy**

|  | Kidney | Prostate | Bladder/Ureter |
| --- | --- | --- | --- |
| Surgery (unilateral nephrectomy, cystoprostatectomy) | 61 | 4 | 10 |
| Surveillance | 6 | 14 | 9 |
| Local resection (TURP/TURBT) |  | 12 | 5 |
| Chemotherapy |  | 2 | 1 |
| Radiotherapy |  | 17 |  |
| Hormone treatment |  | 22 |  |
| Bacillus Calmette-Guerin therapy |  |  | 2 |

TURP-trans-urethral resection of prostate, TURBT-trans-urethral resection of bladder tumour

**Table S2 Association of urological malignancy with all-cause mortality**

**(Cox regression analysis- univariate model)**

|  | Total sample (2637) |  | Matched sample | (640) |
| --- | --- | --- | --- | --- |
| Variable | HR (95% CI) | p-Value | HR (95%CI) | p-Value |
| UM | 1.62 (1.27-2.07) | **0.000** | 1.03 (0.79-1.35) | 0.81 |
| Age | 1.05 (1.04-1.05) | **0.000** | 1.07 (1.06-1.09) | **0.000** |
| Male | 1.17 (1.02-1.34) | **0.02** | 1.30 (0.93-1.83) | 0.12 |
| Caucasian | 2.28 (1.4-3.7) | **0.001** | 1.42 (0.22-11.5) | 0.64 |
| Smoking | 1.7 (1.5-2.02) | **0.000** | 1.49 (1.12-1.97) | **0.005** |
| Alcohol | 0.67 (0.59-0.77) | **0.000** | 0.71 (0.56-0.89) | **0.003** |
| BMI^a^ | 0.99 (098-1.01) | 0.99 | 0.98 (0.95-1.01) | 0.12 |
| Systolic BP | 1.01 (1.0-1.01) | **0.002** | 1.01(1.0-1.01) | 0.049 |
| Diastolic BP | 0.99 (0.98-0.99) | **0.000** | 0.99 (0.98-1.01) | 0.11 |
| Hypertension | 1.69 (1.27-2.35) | **0.000** | 1.27 (0.84-1.91) | 0.25 |
| Diabetes mellitus | 1.85 (1.6-2.09) | **0.000** | 1.39 (1.1-1.75) | 0.005 |
| IHD | 2.01 (1.74-2.31) | **0.000** | 1.19 (0.93-1.54) | 0.16 |
| MI | 2.37 (2.05-2.74) | **0.000** | 1.46 (1.14-1.87) | **0.003** |
| CCF | 2.45 (2.12-2.8) | **0.000** | 1.66 (1.29-2.14) | **0.000** |
| CVA | 1.87 (1.55-2.27) | **0.000** | 1.22 (0.88-1.70) | 0.23 |
| PVD | 1.83 (1.56-2.16) | **0.000** | 1.55 (1.17-2.04) | **0.002** |
| COPD | 1.53 (1.32-1.79) | **0.000** | 1.26 (0.96-1.67) | 0.10 |
| Liver disease | 0.92 (0.61-1.39) | 0.70 | 0.84 (0.39-1.78) | 0.66 |
| RAS blocker | 0.73 (0.64-0.83) | **0.000** | 0.79 (0.63-0.99) | 0.046 |
| Statin | 1.27 (1.12-1.47) | **0.000** | 1.03 (0.81-1.30) | 0.79 |
| ESA | 1.7 (1.47-2.11) | **0.000** | 1.51 (1.10-2.07) | **0.01** |
| uPCR^b^ | 1.0 (1.00-1.01) | **0.68** | 1.01 (1.00-1.01) | **0.002** |
| Creatinine | 1.01 (1.01-1.02) | **0.000** | 1.01 (1.00-1.01) | **0.000** |
| eGFR | 0.97 (0.96-0.97) | **0.000** | 0.97 (0.96-0.98) | **0.000** |

a- BMI missing in 469 of 2477 of total sample and in 112 of 640 of matched sample.

b- missing uPCR values in 265 patients of total sample and 66 patients in matched sample

UM-urological malignancy, BMI-body mass index, BP-blood pressure, HT-hypertension, DM-diabetes mellitus, IHD-ischemic heart disease, MI-myocardial infarction, CCF-congestive cardiac failure, CVA-cerebro vascular accident, PVD-peripheral vascular disease, COPD-chronic obstructive pulmonary disease, RAS-renin angiotensin system, ESA-erythropoietin stimulating agent, uPCR-urine protein creatinine ratio, eGFR-estimated glomerular filtration rate calculated by CKD-EPI.HR-hazard ratio, CI-confidence interval

**Table S3 Association of urological malignancy with end-stage renal disease (Cox regression analysis- univariate model**)

|  | Total sample (2637) |  | Matched sample | (640) |
| --- | --- | --- | --- | --- |
| Variable | HR (95% CI) | p-Value | HR (95%CI) | p-Value |
| Urological malignancy | 1.08 (0.80-1.47) | 0.59 | 1.12 (0.80-1.58) | 0.49 |
| Age | 0.99 (0.98-0.99) | **0.005** | 1.00 (0.99-1.02) | 0.38 |
| Male | 1.29 (1.11-1.51) | **0.001** | 1.75 (1.05-2.88) | 0.03 |
| Caucasian | 0.71 (0.52-0.95) | **0.03** | 0.77 (0.11-5.5) | 0.80 |
| Hypertension | 2.1 (1.50-2.94) | **0.000** | 1.84 (0.98-3.5) | 0.06 |
| Diabetes mellitus | 1.35 (1.1-1.56) | **0.000** | 1.21 (0.89-1.64) | 0.21 |
| IHD | 0.87 (0.71-1.05) | 0.14 | 0.81 (0.56-1.16) | 0.25 |
| MI | 0.86 (0.69-1.07) | 0.18 | 0.86 (0.59-1.24) | 0.43 |
| CCF | 1.07 (0.88-1.31) | 0.47 | 0.99 (0.68-1.44) | 0.97 |
| CVA | 1.25 (0.98-1.59) | 0.06 | 1.10 (0.69-1.74) | 0.67 |
| PVD | 1.15 (0.94-1.41) | 0.18 | 1.43 (0.98-2.07) | 0.06 |
| eGFR | 0.91 (0.90-0.91) | **0.000** | 0.89 (0.87-0.90) | **0.000** |

IHD-ischemic heart disease, MI-myocardial infarction, CCF-congestive cardiac failure, CVA-cerebro vascular accident, PVD-peripheral vascular disease, eGFR-estimated glomerular filtration rate calculated by CKD-EPI.
