# Supplementary figures and images for: Associations of urological malignancies with renal progression and mortality in advanced chronic kidney disease: a propensity-matched cohort study
Source: BMC Nephrol. 2020 May 29;21:202. doi: 10.1186/s12882-020-01859-w (PMC7257121; doi:10.1186/s12882-020-01859-w)

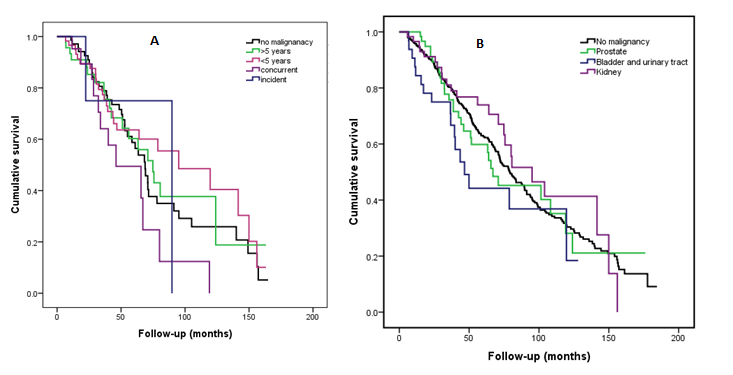

Supplement: Supplementary file 2 — Additional file 2: Figure S1. Kaplan-Meier curve for all-cause mortality in the matched sample (A: comparison between groups split based on date of cancer occurrence prior to recruitment; B: comparison between groups split based on site of cancer). [file 12882_2020_1859_MOESM2_ESM.png]
